# Triclosan has a robust, yet reversible impact on human gut microbial composition *in vitro*

**DOI:** 10.1371/journal.pone.0234046

**Published:** 2020-06-25

**Authors:** Karley K. Mahalak, Jenni Firrman, Jung-Jin Lee, Kyle Bittinger, Alberto Nuñez, Lisa M. Mattei, Huanjia Zhang, Bryton Fett, Jamshed Bobokalonov, Gustavo Arango-Argoty, Liqing Zhang, Guodong Zhang, Lin Shu Liu

**Affiliations:** 1 United States Department of Agriculture, Dairy and Functional Foods Research Unit, Agricultural Research Service, Eastern Regional Research Center, Wyndmoor, Pennsylvania, United States of America; 2 Division of Gastroenterology, Hepatology, and Nutrition, Children’s Hospital of Philadelphia, Philadelphia, Pennsylvania, United States of America; 3 Department of Computer Science, Virginia Tech, Blacksburg, Virginia, United States of America; 4 Department of Food Science, University of Massachusetts, Amherst, Massachusetts, United States of America; 5 Molecular and Cellular Biology Graduate Program, University of Massachusetts, Amherst, Massachusetts, United States of America; Universidade Catolica Portuguesa, PORTUGAL

## Abstract

The recent ban of the antimicrobial compound triclosan from use in consumer soaps followed research that showcased the risk it poses to the environment and to human health. Triclosan has been found in human plasma, urine and milk, demonstrating that it is present in human tissues. Previous work has also demonstrated that consumption of triclosan disrupts the gut microbial community of mice and zebrafish. Due to the widespread use of triclosan and ubiquity in the environment, it is imperative to understand the impact this chemical has on the human body and its symbiotic resident microbes. To that end, this study is the first to explore how triclosan impacts the human gut microbial community *in vitro* both during and after treatment. Through our *in vitro* system simulating three regions of the human gut; the ascending colon, transverse colon, and descending colon regions, we found that treatment with triclosan significantly impacted the community structure in terms of reduced population, diversity, and metabolite production, most notably in the ascending colon region. Given a 2 week recovery period, most of the population levels, community structure, and diversity levels were recovered for all colon regions. Our results demonstrate that the human gut microbial community diversity and population size is significantly impacted by triclosan at a high dose *in vitro*, and that the community is recoverable within this system.

## Introduction

Triclosan (TCS) has been used as a general antimicrobial in many consumer goods, including soaps, toothpaste and other personal care products, since the 1960s. In 2016, the FDA issued a ban on TCS and 18 other antimicrobials that were being used in consumer goods [1]. This decision was mainly based on recent studies which showed that soaps containing TCS did not provide additional benefits when compared with plain soaps without antimicrobials; therefore the high volume, low-value use of TCS in over-the-counter handwashing products was not further allowed in the USA [1].

TCS is bacteriostatic at low doses (0.01–0.1 mg/L), and bactericidal at high doses (1mg/L or higher) [2–4]. Initially, due to its ability to work against a diverse range of microbes, it was thought that use would not contribute to the antibacterial resistance problem, which is the reason TCS was used so widely in consumer goods. However, it was later found that *E*. *coli* was able to develop resistance to TCS [5, 6]. Through further exploration, it was discovered that TCS functions through disruption of fatty acid synthesis via inhibition of Enoyl Acyl Carrier Protein Reductases (ENRs). TCS binds non-covalently to NAD^+^, forming a stable FabI-NAD^+^-Triclosan complex thereby preventing the continuation of fatty-acid synthesis [7]. The inability to continue fatty acid synthesis disrupts the growth of cell membranes, leaving bacteria and fungi unable to grow, divide, and propagate. Recent studies have shown that use of TCS is associated with enrichment of triclosan resistance genes, which are passed on through horizontal gene transfer [8, 9]. A study published in 2019 revealed an unfortunate side effect of a reduction of antibiotic efficacy and an increase in persister cells, indicating that previous dosing with TCS rendered a bacterial population more resistant to a variety of antimicrobial compounds both *in vivo* and *in vitro* [10].

TCS is absorbed by mice and humans through skin and mucus membranes, including that of the gastrointestinal tract [11–18]. In 2003–2004, a study on urinary concentrations of TCS in a mixed human population discovered that the majority of the population over 6 years of age had detectable levels of TCS in their urine, and that this concentration increased with increasing income categories [19]. In a Swedish study of human milk, TCS was found in detectable levels in 3 out of 5 samples in a milk bank[20]. This absorption is attributed to the personal care products that people use on their skin as well as the TCS found in some toothpaste brands. However, due to TCS in the environment from waste water treatment plants and bioaccumulation in plant and animal life that are consumed by humans, it is likely that humans are obtaining these amounts of TCS due to ingestion [20–24].

Extensive use of TCS over the past several decades has made the compound and its derivatives such as M-Triclosan, prevalent in the environment [12, 21]. That, along with the increase in Inflammatory Bowel Disease (IBD) in youth populations around the world, has raised concerns over how TCS and other antimicrobials may impact the gut microbial community[25]. In mice, TCS induced colonic inflammation was shown to be dependent on the presence of a gut microbial community. The consumption of TCS was also shown to alter the gut microbial community alpha and beta diversity significantly, specifically reducing the Bifidobacterium population, which promote immune tolerance [26]. A recent study with zebrafish showed similar results with respect to a significant decrease in gut microbial diversity within days of TCS exposure, with decreasing stability of the microbial community over time [27]. A 13-week mouse study showed that consumption of TCS in drinking water caused significant changes in the gut microbial community as well as an increase in bacterial genes involved in antimicrobial resistance, heavy metal resistance and those involved in the stress response [28]. While these studies address the impact of TCS on the gut microbiota *in vivo*, the analyses were only performed after completion of TCS treatment, and there was no delineation between colon regions for these studies. These studies also did not address what occurs to the gut microbial community over the course of the treatment. The present study attempts to address these limitations by using an *in vitro* system to observe changes in the community over the course of treatment with TCS. Additionally, this study evaluates the effect of TCS on the regionally distinct microbial communities that develop within the ascending, transverse, and descending colon regions [29].

The understanding that humans readily absorb TCS and are able to pass Triclosan through breastmilk has led to a need to explore the impact of Triclosan on the human gut microbiome [30]. In this study, we analyzed the effects of TCS on the gut microbiota using a Triple Simulator of the Human Intestinal Microbial Ecosystem (Triple SHIME®). Here, we combined 16S RNA sequencing with short-chain fatty acid analysis, bacterial community diversity analysis, and shotgun sequencing to determine the impact of both a low and high dose of Triclosan on the gut microbial community as a whole *in vitro*. This system allowed us to track the changes in the human gut microbial community over time, as well as in three different regions of the colon, the ascending (AC), transverse (TC), and descending (DC) regions. Our research revealed significant changes over time to the gut microbial community in response to different doses of TCS. We also show that the community population and diversity are able to return to nearly pre-treatment levels after a 2 week recovery period. To the best of our knowledge, this is the first report on the impact of TCS on the *in vitro* human gut microbial community structure and function over a period of time.

## Materials & methods

### Materials

Materials for the basic running of the Triple SHIME® were obtained and used as described previously [31]. The medium used in the bioreactors for this experiment was purchased from Prodigest (Ghent, Belgium) as the Defined Medium (DM) [31]. Pancreatic Juice was made from 12.5 g/L NaHCO_3_ (Sigma-Aldrich, Saint Louis, MO), 6 g/L Bile Salts (BD, Franklin Lakes, NJ), and 0.9g/L pancreatin (Sigma-Aldrich, Saint Louis, MO). Mucin-Agar was prepared as described previously using 5% type II porcine mucin (Sigma-Aldrich) and 1% bacterial agar into sterile MilliQ water [31].

Screened, healthy, homogenized human fecal sample was obtained from OpenBiome (www.openbiome.org) as described previously [31, 32]. The sample was harvested from an American with an average Body Mass Index (BMI), between the ages of 21 and 45 years of age, who had been free of antibiotics for at least 1 year, and consumed a typical western diet. This samples was selected at random from a pool of consumers meeting these criteria. The same homogenate was used to inoculate all three systems simultaneously for each part of the experiment. Triclosan (Sigma-Aldrich, Saint Louis, MD) was dissolved by mixing into Olive Oil at 0.038g/mL for the low dose phase and at 0.38 g/mL for the high dose phase.

### *In vitro* experiment using Triple SHIME®

The Triple SHIME® (Prodigest, Ghent, Belgium) was set up and run similar to the TWINSHIME® system following the manufacturers guidelines with slight modifications [31, 32].—. In the Triple SHIME® set up, shown in S1 Fig, a single bioreactor is used for the stomach and small intestine regions for all three colon systems. All three systems have three regions of the colon represented, the ascending colon (AC), transverse colon (TC) and the descending colon (DC). All colon regions contained 60 mucin carriers to mimic bacterial growth on the mucosal surface in the colon [31, 32]. Temperature was maintained at 37°C over the course of the experiment and an anaerobic environment was maintained by using sterile nitrogen flow and sealing the vessels. pH was maintained using 0.5M NaOH and 0.5M HCL. PH of the vessels are as follows: 2 for the Stomach, 6.6 for the Small Intestine, 5.75 ±0.15 for the AC, 6.12±0.125 for the TC, and 6.75±0.15 for the DC [31, 32]. This unique system allows for the sequential movement of feed through the simulated digestive tract, from the stomach, into the small intestine, and sequentially into each of the three colon regions 3 times a day. This, along with the ability to cultivate both lumenal and mucosal microbial communities allows for this system to be a comprehensive study of the human digestive tract *in vitro*.

This experiment was run for a total of 49 days. The system was inoculated with homogenized human fecal sample at 5% reactor volume and allowed to stabilize for 21 days. Day 22-Day 28, a low dose of Triclosan, (0.019g two times a day, dissolved in Olive Oil) was injected at feeding time for the two experimental reactor systems (AC2 & AC3), while 0.5 mL of Olive Oil were injected into the AC1 bioreactor as a control. Day 29–34, a high dose of Triclosan dissolved in Olive Oil (0.19g two times a day) was injected with injected olive oil serving as a control. Day 35–49 was the recovery period wherein only normal feeding cycles were completed. Samples were collected on a regular basis throughout the experiment. Every sampling, lumenal fluid was centrifuged at 5000 x *g* for 10 m at 4°C. Supernatant was filtered through a 0.22μm PES filter to create a bacterial free supernatant and stored at -80°C for SCFA analysis. The bacterial pellet was also stored at -80°C for DNA extraction purposes. Mucosal samples were harvested by removing ½ of the mucin carriers from each vessel every second day during the experiment, and the mucin agar was aliquoted into tubes at 0.25–0.5g and stored at -80°C for DNA extraction purposes.

### 16S rRNA gene qPCR

Total microbial DNA extraction of both lumenal and mucosal samples were performed using the CTAB DNA extraction method, as described previously [33]. DNA concentrations were quantified using a Nanodrop and stored at -80°C until they were shipped for sequencing. Bacterial abundances were measured by a quantitative PCR (qPCR) assay targeting the V1-V2 region of the 16S rRNA gene. Each sample was assayed in triplicate reactions composed of 6.25 μl of 2X TaqMan(TM) Fast Universal PCR Master Mix (Thermo Fisher Scientific), 0.05 ul of each primer at 100 μM, 0.01 μl of probe at 100 μM, 1.14 μl of molecular grade water, and 5 μl DNA diluted 1:100, 1:200, or 1:1000 in water. Cycling conditions were as follows: 1 cycle at 95°C for 20 s; 40 cycles of 95°C for 3 s and 60°C for 30 s. Signal was collected during the elongation step at 60°C. A standard curve was generated from a plasmid containing the full length 16S rRNA gene from Streptococcus. The sequences of the primers and probe are provided in Table 1 [34].

**Table 1:** Primer and probe sequences.

BSF8	AGAGTTTGATCCTGGCTCAG
BSR357	CTGCTGCCTYCCGTA
Probe	/56-FAM/TAA +CA+C ATG +CA+A GT+C GA/3BHQ_1/

All nucleotides are listed 5’ to 3’ and purchased from IDT. The + in the probe indicates locked nucleic acids

### Microbiome 16S rRNA marker gene sequencing

Lumenal samples extracted using the CTAB DNA extraction method were stored at -80°C until library preparation. To improve library yield, fresh aliquots of mucosal samples were extracted from approximately 300 μl of SHIME mucosal contents using the Qiagen DNeasy PowerSoil kit. DNA concentration was quantified using Quant-iT PicoGreen dsDNA Assay Kit (Thermo Fisher Scientific). There were 310 samples in total that were extracted and sequenced. The V1-V2 region of the 16S rRNA marker gene was amplified using barcoded primers and AccuPrime™ *Taq* DNA Polymerase, High Fidelity (Thermo Fisher Scientific) in quadruplicate PCR reactions. Each PCR reaction contained 0.19 μl of polymerase, 2.5 μl of 10x PCR Buffer, 5 μl of each primer at 2 μM, 2.5 μl DNA, and 9.81 μl of molecular grade water in a total volume of 25 μl. Cycling conditions were as follows: 1 cycle of 95°C for 5 m; 20 cycles of 95°C for 30 s, 56°C for 30 s, and 72°C for 90 s; 1 cycle of 72°C for 8 m. Replicate reactions were combined and then libraries were quantified using the Quant-iT PicoGreen dsDNA Assay Kit (Thermo Fisher Scientific). Each library was pooled in equimolar amounts and then the pool was purified using SPRI beads (GE Healthcare). DNA sequencing was performed on a MiSeq instrument (Illumina) using 2 × 250 bp chemistry. Extraction blanks and DNA-free water were processed in parallel to as negative controls for environmental and reagent contamination. Positive controls, consisting of eight artificial 16S gene fragments, were also included.

### Shotgun metagenomic library construction and sequencing

Lumenal samples previously sequenced on the Illumina MiSeq were selectively used to construct shotgun metagenomic libraries. Libraries were constructed using an Illumina Nextera XT Library Prep Kit (Illumina). The concentration and size of the libraries were measured using the Quant-iT PicoGreen dsDNA Assay Kit (Thermo Fisher Scientific) and the HS NGS Fragment Kit for the Fragment Analyzer (Agilent), respectively, and then equimolar quantities were pooled together. DNA sequencing was performed on a HiSeq 2500 instrument using 2 x 125 bp chemistry (Illumina). DNA extraction blanks and DNA-free water were included as negative control samples to assess environmental and reagent contamination. Laboratory-generated mock communities consisting of DNA from *Vibrio campbellii* and Lambda phage were included as positive control samples.

### Bioinformatics

16S RNA V1V2 region sequencing data was processed and analyzed using the QIIME2 pipeline as described previously [35]. The QIIME2 implementation of DADA2 [PMID 27214047] was used for sequence quality filtering and taxonomy was assigned using a Naïve Bayes classifier trained on the Greengenes 13_8 99% OTUs. For diversity metrics such as Faith diversity and UniFrac distances, a rooted phylogenetic tree was generated. First of all, a multiple sequence alignment was performed using MAFFT [PMID 23329690] and high variable positions were masked to reduce noise in a resulting phylogenetic tree. Secondly, a mid-point rooted tree was generated using FastTree [PMID 20224823]. For shotgun metagenomics data, the 125bp paired-end sequence reads were quality-filtered and trimmed to remove adapter sequences using Trimmomatic [PMID:24695404]. Reads attributed to the human host genome (version hg38) were removed using Burrows-Wheeler Alignment [PMID: 19451168]. Taxonomic annotations were generated with Kraken [PMID: 24580807], using the standard Kraken database with all complete bacterial, archaeal, and viral genomes in NCBI RefSeq.

### Short chain fatty acid analysis by GC-MS

The SCFA method involved preparing calibration standard mixtures of the straight chain SCFA (Acetic Acid, Proprionic Acid, Butyric Acid, Pentanoic Acid, and Hexanoic Acid) and the branched chain SCFA (BSCFA) (Isobutyric Acid, 2-Methylbuyric Acid, 3-Methylbutyric Acid, 2-Methylpentanoic Acid, 3-Methylpentanoic Acid, and 4-Methylpentanoic acid). A calibration curve for each compound was established from 1.5 ppm to 5000ppm using the internal standard 2-Methylhexanoic Acid. All the standards were analytical grade and purchased from Sigma Aldrich (Saint Louis, MO).

The filtered lumenal samples were thawed at 40°C for 30 minutes and then extracted using liquid-liquid extraction with diethyl ether (1:4 v:v). 1 μl of the SCFA and BSCFA extracts were injected into the 260°C injection port of a GC/MS (Shimadzu QP2010 Ultra; Shimadzu, Columbia, MD) with a 30m Stabilwax-DA column (0.25mm ID, 0.25μm; Restek Corporation, Bellefonte, PA, USA). Separation of the SCFA was achieved with the following settings: a split ratio of 1:20 and a flow rate of 1.00 ml/min of helium, oven temperature ramp starting at 125°C and held for 1 min and then ramped to 250°C over 12.5 min. The interface temperature between the GC and MS was held at 250°C and the ion source temperature of the MS was 220°C.

The average total SCFA is the summation of all SCFAs measured in each intestinal region after stabilization, and the average BSCFA is the summation of all branched SCFAs measured in each intestinal region after stabilization (Days 20, 23, 27, 30, 34, 38, 41, and 43). At each time point 3 x 1 ml samples were taken from each bioreactor, each sample was extracted three times, each extraction was measured three times on GC/MS.

### Bile acid analysis by HPLC-MS

Bile acids were analyzed using a Nano-Acquity (Waters Co. Milford, MA) high-pressure liquid chromatograph (HPLC) equipped with a Halo C18, 2.7μm column (1x150 mm) (Advanced Materials Technology Inc. Wilmington, DE). Column was set at 40°C and run at 60μL/m using solvent A: water 5 mM ammonium formate and 0.012% formic acid and solvent B: methanol 5 mM ammonium formate and 0.012% formic acid, with the following gradient: initial time to 1m 30% solvent A and 70% solvent B; ramped with a linear gradient to 5% A and 90% B, returning to the initial conditions after 15m with 10m of equilibration time between injections.,.

The HPLC column was directed to a Synapt G1 quadrupole time of flight mass spectrometer (Waters Co.) equipped with an electrospray probe operated in negative mode set a 2.5Kv capillary voltage and 300°C with nitrogen flow of 300 L/m. The cone voltage was set at 40 V and the collision energy was set a 6 eV to avoid fragmentation of the product analyzed.

Samples were divided in three fractions of 100μl and prepared for HLPC/MS analysis by adding 100μL of a solution of cholanic acid (0.4 mg/mL) in methanol, followed by the addition of 400μL of acetone and centrifuged for 1m. After centrifugation, 100μL from the supernatant were mixed with 400μL of acetonitrile for injection (1 μL) into the HPLC. Data analysis and quantification were performed with MassLynk software V 4.1 (Waters Co.) and Microsoft Excel to correlate the area of the peaks of the bile acids with the internal standard using the average of three injections.

### GRiD analysis

Growth Rate InDex (GRiD) analysis was performed on the data from shotgun sequencing using methods described by Emiola & Oh 2018 to determine growth rates of particular bacterial species of interest [36]. Growth rates of 20 selected bacterial organisms were determined from lumenal samples taken from the ascending, transverse, and descending colon regions. The growth rates of the two experimental groups were combined for comparison to control. For each region, the Pearson Correlation coefficient was computed between the control and treated groups. A high positive correlation indicates that the TCS treatment does not impact growth patterns whereas a high negative correlation indicates that TCS treatment has an adverse effect on the growth rate patterns. Wilcoxon signed-rank test was conducted to determine whether TCS treatment has a significant effect on the growth rate of treated groups, but due to low sample number none of the tests is significant.

## Results

### TCS significantly impacts alpha diversity of the gut microbiota *in vitro*

**Fig 1:**
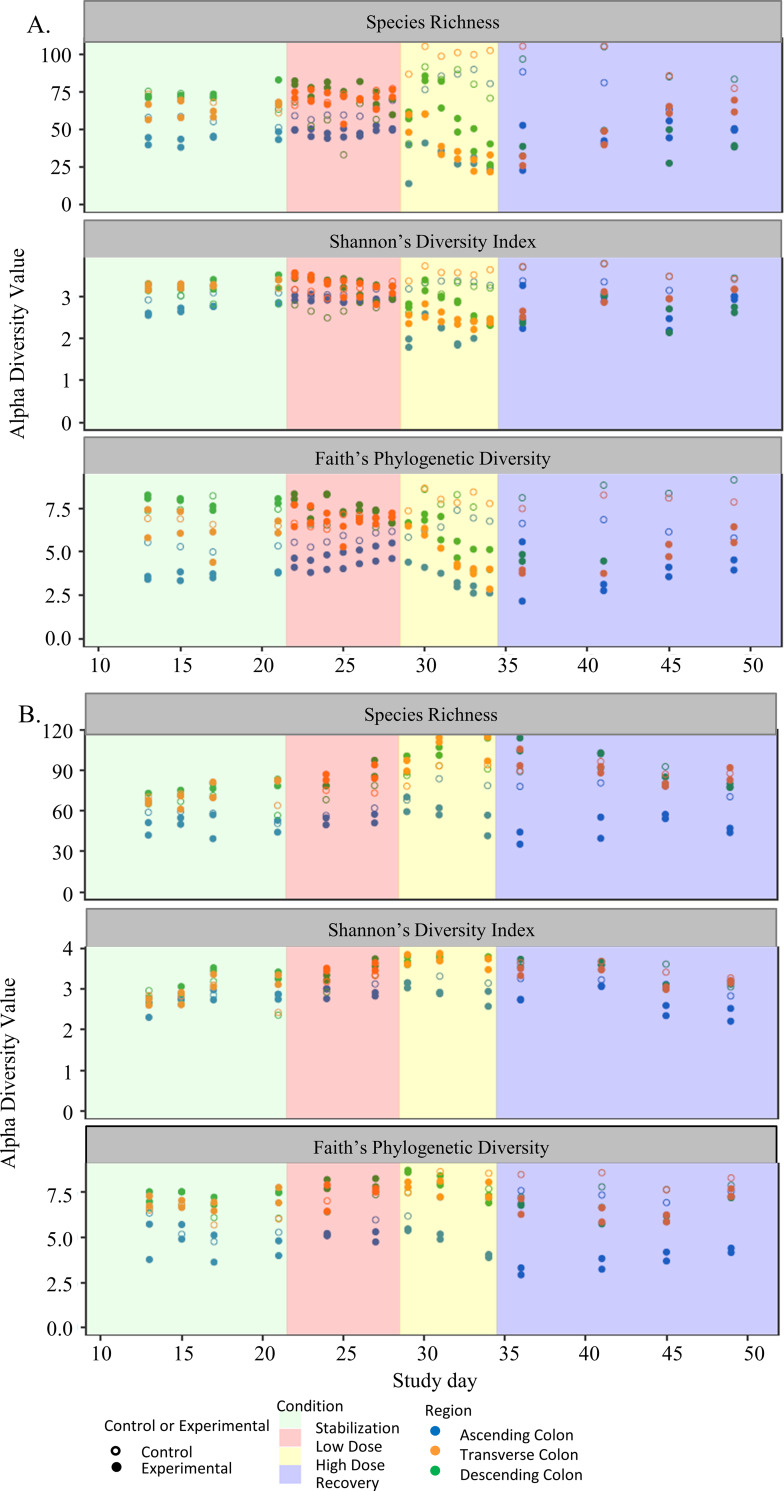
Alpha diversity based on 16S RNA gene sequencing data over time. Top frame is based on species richness, Middle frame is based on the Shannon diversity index, bottom frame is based on Faith’s phylogenetic diversity index.

After stabilization of a gut microbial community using a Triple SHIME® configuration, we treated the community with a low dose of triclosan (TCS) for 1 week, immediately followed by a high dose of TCS for 1 week and finished with a 2 week recovery period. The effects of triclosan on the gut microbiota was assessed in terms of alpha diversity (Fig 1). Statistical analysis was performed on the data using ANOVA, significance was determined at p<0.05. With respect to species richness (top panel), which indicates the number of different species present, the addition of TCS at a low dose had minimal effect, however, richness changed significantly during the high dose period for both the lumenal (Fig 1A) and mucosal (Fig 1B) phases. In the lumenal phase, we see a consistent decline in species richness throughout the high dose period across all three colon regions, and a subsequent rise toward control levels over the 2 week recovery period, though control levels were not reached at the end. In the mucosal phase, we only observe a consistent decline in species richness in the AC region that never recover back to control levels. In the TC and DC regions, there is an increase in species richness during the high dose phase. In Fig 1, we note that the difference between the control and experimental regions are statistically significant (p value <0.05). There is a significant difference between the control and all three experimental conditions (low dose, high dose, and recovery) (p value <0.05).

We used Shannon’s Diversity Index to take into account both the abundance and evenness of the species that are present in each sample. In the middle panel of Fig 1A, we saw a decrease in diversity in the lumenal phase over the course of the high dose period with both the AC and DC colon regions, whereas the TC region remained stable throughout. We also observed a return towards the control at the end of the recovery period. For the mucosal phase, we saw the most impact occurring in the AC region during the high dose TCS treatment, whereas the TC and DC regions continue to remain stable through that treatment period (Fig 1B, middle panel). These diversity levels appear to lower during the recovery period compared with the high dose period, and don’t quite reach the same level as control in the AC at the end of the recovery period. As with the species richness, we see a significant difference between the control and experimental samples in terms of colon regions, as well as between the control and experimental samples with respect to experimental condition.

Finally, we used Faith’s Phylogenetic Diversity (FPD) Index (Fig 1, lower panel) to show diversity levels based on ASV distances instead of species, allowing us to look at diversity at a sub-species level. Using this measure, we see that the lumenal phase samples (Fig 1A, lower panel) have little response to the low dose period but decrease significantly in diversity during the high dose period in all three regions of the colon. The FPD does increase towards control levels during the recovery period but never reaches complete recovery. The mucosal phase also shows most of the impact of TCS on the Ascending colon region during the high dose period. Similarly to the other measures of alpha diversity, TCS has little impact on the Transverse and Descending colon regions over the course of high dose treatment, though they do show a decrease in FPD towards the end of the high dose period that continues into much of the recovery period. In this measure of diversity as well, there is a significant difference between the control and experimental groups with respect to both the colon region and the experimental condition in both the lumenal and mucosal phases.

### Beta diversity of the *in vitro* gut microbiota is significantly impacted by TCS

**Fig 2:**
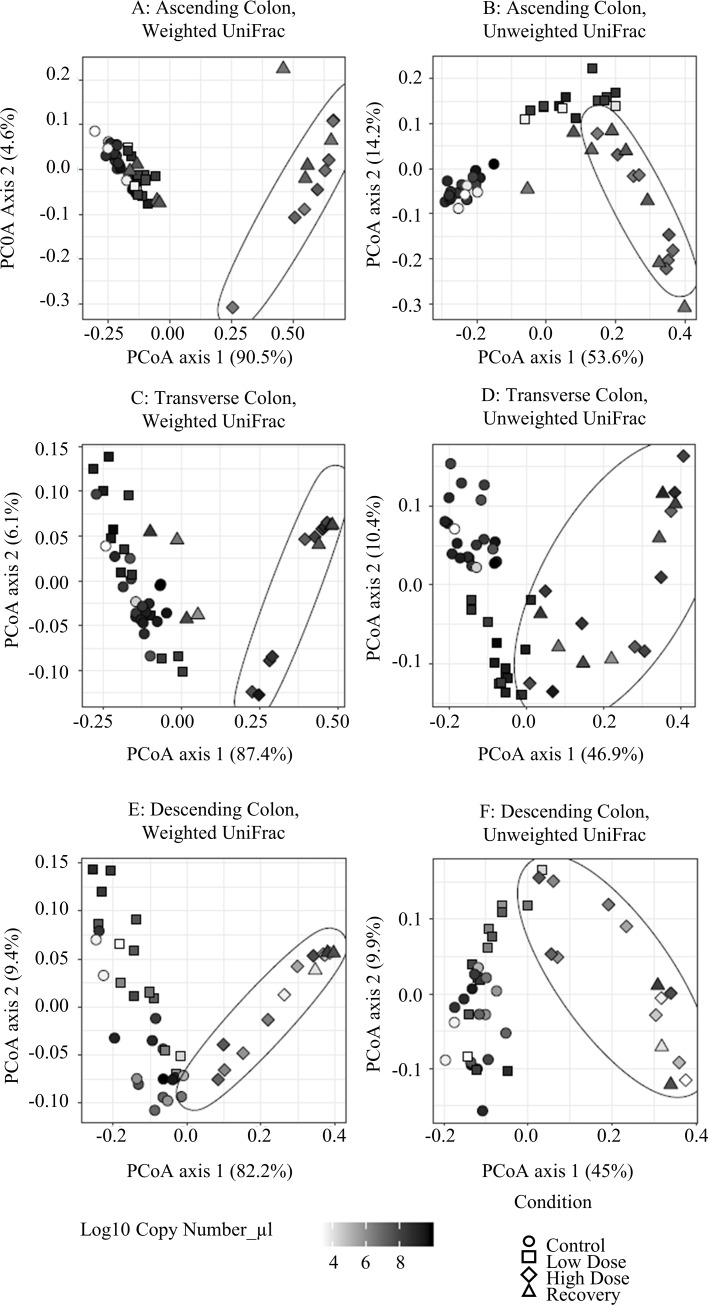
Beta diversity of lumenal phase combined with copy number (qPCR). High dose samples are inside the ellipses. A) Weighted UniFrac, ascending colon. B) Unweighted Unifrac, ascending colon. C) Weighted Unifrac, transverse colon. D) Unweighted Unifrac, transverse colon. E) Weighted UniFrac, descending colon. F) Unweighted Unifrac, descending colon.

**Fig 3:**
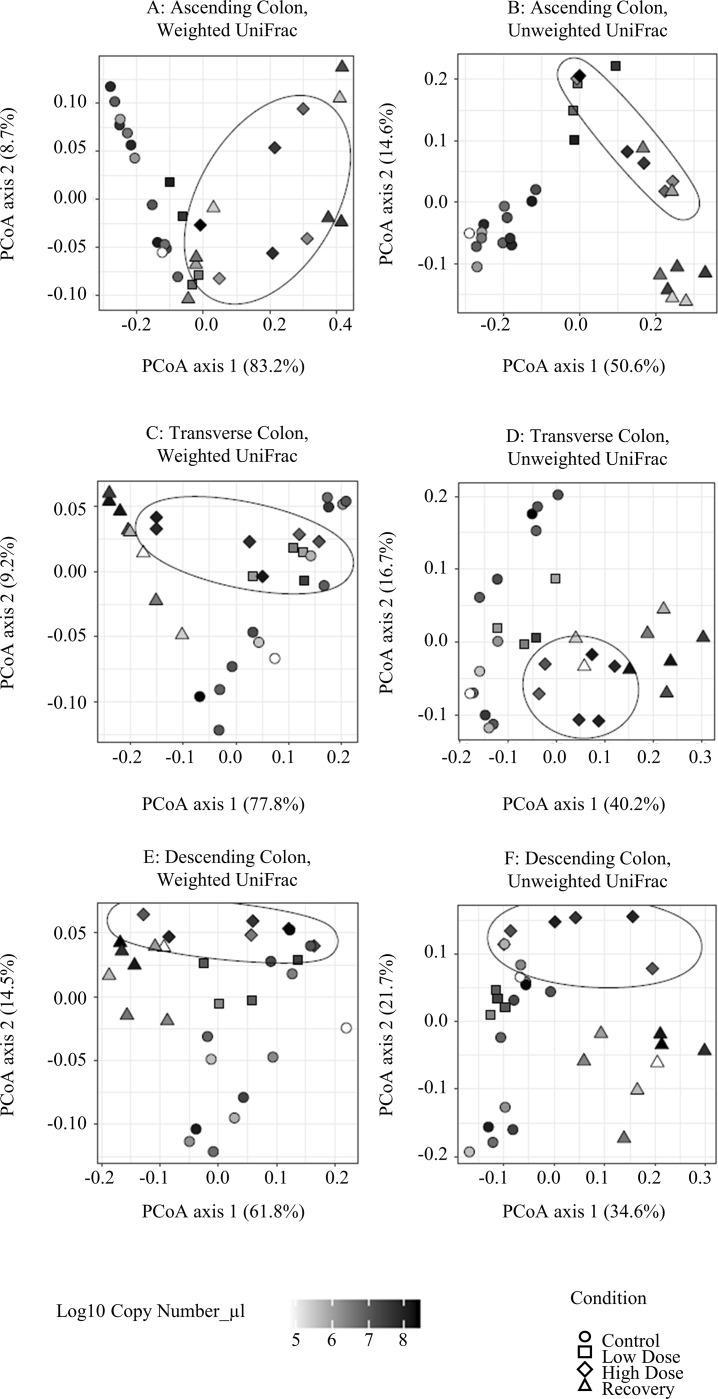
Beta diversity of mucosal phase combined with copy number (qPCR). A) Weighted UniFrac, ascending colon. B) Unweighted Unifrac, ascending colon. C) Weighted Unifrac, transverse colon. D) Unweighted Unifrac, transverse colon. E) Weighted UniFrac, descending colon. F) Unweighted Unifrac, descending colon.

Beta diversity of the samples was measured using Principle Coordinates Analysis (PCoA) to visualize the similarity between samples using weighted and unweighted UniFrac distance measurements. The left column of Figs 2 & 3 illustrates the results of the weighted UniFrac Distance measurements, or those that are based on the abundance of the observed organisms, whereas the right column of Figs 2 and 3 show the results of the unweighted measurements that are based on the presence or absence of each organism. We combined our qPCR data with these charts to show how the density of the bacterial community contributes to its diversity over the course of the treatment with TCS.

For the Weighted UniFrac analysis of the Lumenal phase (Fig 2, left column), we observed a clear distinction between the high dose samples and the control & low dose samples in all three colon regions. Unsurprisingly, we see that the community takes time to recover. Combined with copy number, we can see that this change in beta diversity occurs as the bacterial population decreases beyond a particular level, yet moves back towards control as the population recovers. In the Unweighted UniFrac charts, we observe qualitative changes in the bacterial diversity. For the AC (Fig 2B), we observe a very clear clustering effect in the control group compared with all of the experimental condition groups. The TC and DC (Fig 2D & 2F), however, do not have such a tight clustering effect with respect to the high dose compared with the other conditions.

For the Weighted UniFrac analysis of the mucosal phase (Fig 3, left column), we found that the clustering of the high dose samples was not as closely-knit as those of the lumenal phase. However, the high dose samples were separated from the control groups in the AC much more clearly than in the TC or DC regions. For the Unweighted UniFrac analysis (Fig 3, right column), we see a similar pattern where the high dose period is not observed as being quite as distinct from the other experimental conditions as what we observe in the lumenal phase samples. These results indicate that the lumenal phase is more strongly impacted by treatment with TCS when compared to the mucosal phase, and that the AC region is the most impacted by this treatment.

### TCS exposure changes bacterial community composition

Relative abundance of the bacterial community at the family level was determined using 16S rRNA gene sequencing for both lumenal and mucosal phase samples. The stabilization period aligned with the control group, showing a consistent relative abundance in all the communities of all three colon regions.

For the lumenal phase samples (S2 Fig), in the AC during the low dose phase of TCS treatment, there was a significant decrease in *Lachnospiraceae Clostridium* and *Fusobacteriaceae Fusobacterium* genera, however in the TC and DC regions there was a significant decrease in *Synergistaceae Cloacibacillus* and *Alcaligenaceae Sutrella* had a significant decrease in proportional abundance in the DC. During the high dose phase, however, there were significant decreases in the *Bacteroides*, *Clostridium*, *Parabacteroides*, *Fusobacterium* and more genera. The majority of these differences were resolved during the recovery period of the experiment. For the mucosal samples (S3 Fig), there were significant decreases in proportional abundance in *Bacteroidaceae Bacteroides*, and *Rikenellacaea* genera during the high dose period between the control and experimental AC regions. *Enterobacteriacaea Trabulsiella* exhibited a significant decrease in proportional abundance in the AC region during the low-dose treatment phase and the *Sutterella* genera exhibited a significant decrease in proportional abundance in the DC during the recovery period when compared with control.

**Fig 4:**
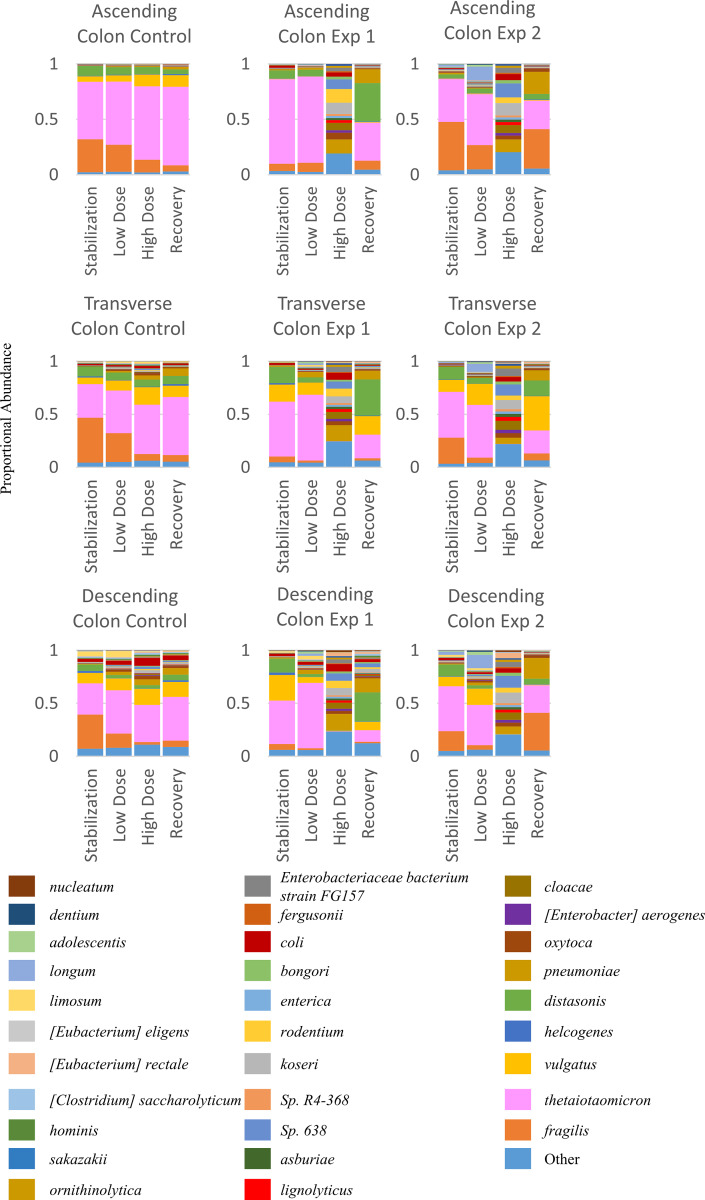
Bacterial community population data based on shotgun metagenomic sequencing at the species level. Unit 1 (A1, T1, D1) on the left panel are the control groups. Middle and right panels are the experimental groups (n of 2).

Since our analysis showed such a large change in bacterial abundance, we performed shotgun sequencing on a select number of lumenal samples to get a more detailed view of the results. In Fig 4, we show the proportional changes that occur in the community over the course of the experiment at the species level. We saw a similar change with shotgun sequencing as with 16S RNA sequencing, showing a strong decrease in several *Bacteroidetes* species, and an increase in *Proteobacteria* species. The most extreme change seen was in *Bacteroides thetaiotaomicron* (pink), whose population dropped drastically after the high dose period, but showed an ability to recover towards normal population levels during the recovery period. We see similar, though less drastic drops in population in other *Bacteroidetes* species, such as *Bacteroidetes fragilis* (orange). Conversely, we see a proportional increase in abundance with many of the *Proteobacteria* species during the High Dose period, with a reduction back towards control population levels during the recovery period. However, one species, *Klebsiella pneumoniae* (Dark Yellow), did not show any indication of a decrease to normal levels. This is not too surprising because it’s a species known to have multi-drug resistant strains. A notable difference between those species that decline in population compared with those that increase in population with a high dose of TCS is that those that thrive in this environment are facultative anaerobes, and those that do not thrive are obligate anaerobes.

### Common genes impacted by TCS

The shotgun sequencing was also used to explore which genes were present or absent in response to TCS, based on genetic potential. We found that genes that were absent in response to a high dose of TCS were mostly those involved in DNA replication and repair. Conversely, genes that were present in higher quantities in response to a high dose of TCS were those involved in transmembrane transport (S4 Fig). The peptide/nickel transport system substrate-binding protein was a gene that had a strong presence during the high dose treatment of TCS.

**Fig 5:**
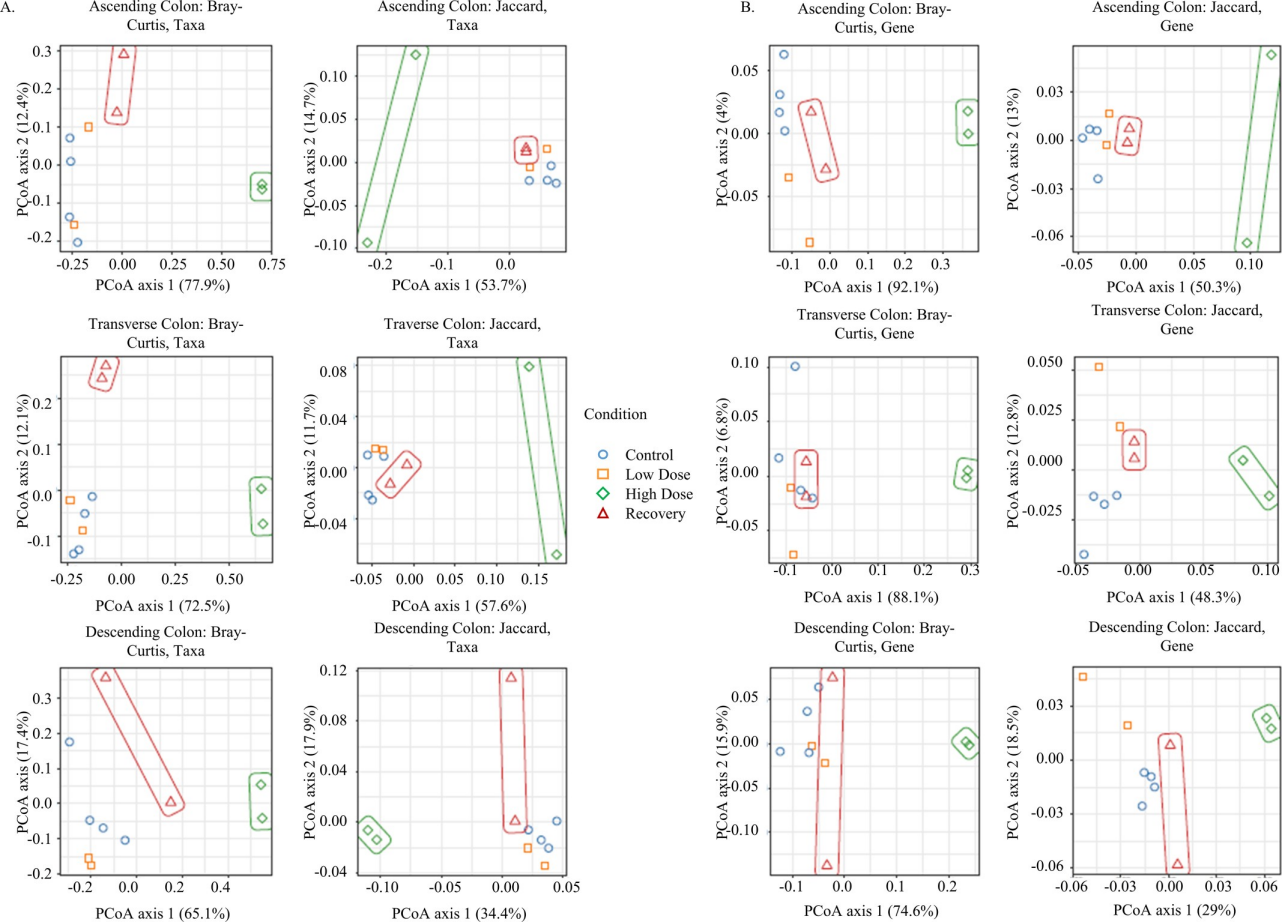
Gene analysis from shotgun sequencing. A) Taxa based PCoA plots. Bray-Curtis (left) is based on the proportion/composition of the taxa. Jaccard distance (right) is based on the presence/absence of the taxa B) Beta diversity based on KEGG Ortholog (KO) composition, meaning the presence/absence of gene function is used to measure the Bray-Curtis distance.

Principal Coordinates Analysis (PCoA) was plotted to allow the visualization of the distance measured between samples (Fig 5A and 5B). In Fig 5A, the beta diversity plots are based on the taxa present in the samples, the Bray-Curtis plots on the left describe the beta diversity in terms of abundance of the components, where the Jaccard distance based plots on the right describes only the presence or absence of specific taxa in each sample. If there was a large difference between the Bray-Curtis plots compared with the Jaccard Index plots, it would indicate that there is a different in the type of taxa that are impacted by TCS, the Jaccard Index being a test more sensitive to the rare taxa. However, the results we see indicate that there is not a real difference in the impact of TCS on the bacteria based on their initial abundance in the community population. In Fig 5B, we used KEGG orthology (KO composition) to look at the similarity between samples in terms of gene function instead of taxa. In all cases, regarding taxa-based or KO composition (a.k.a. gene function) based analysis, the high dose samples (shown in green) were maximally different from other samples. This is not surprising given the other results discussed above, where the high dose samples are consistently the most significantly different from control samples in terms of community composition and diversity.

Additionally, we explored the impact of TCS on the amount of enoyl-acyl carrier proteins available in the system. As mentioned earlier, these proteins are involved in fatty acid synthesis and missense mutations in these proteins (Such as FabI) have conferred resistance to TCS by disallowing the formation of a TCS-NAD+-FabI ternary complex [5, 7]. Looking at the population levels (S5 Fig), we see a drop in the proportional abundance of this gene with two of the related proteins (K00208, and K02371). One of these carrier protein genes (K00209) shows no change in proportional abundance over the course of the experiment, and the other (K10780) appears to grow in abundance.

### TCS exposure drastically reduces production of SCFAs and bile acids in the gut microbial community

**Fig 6:**
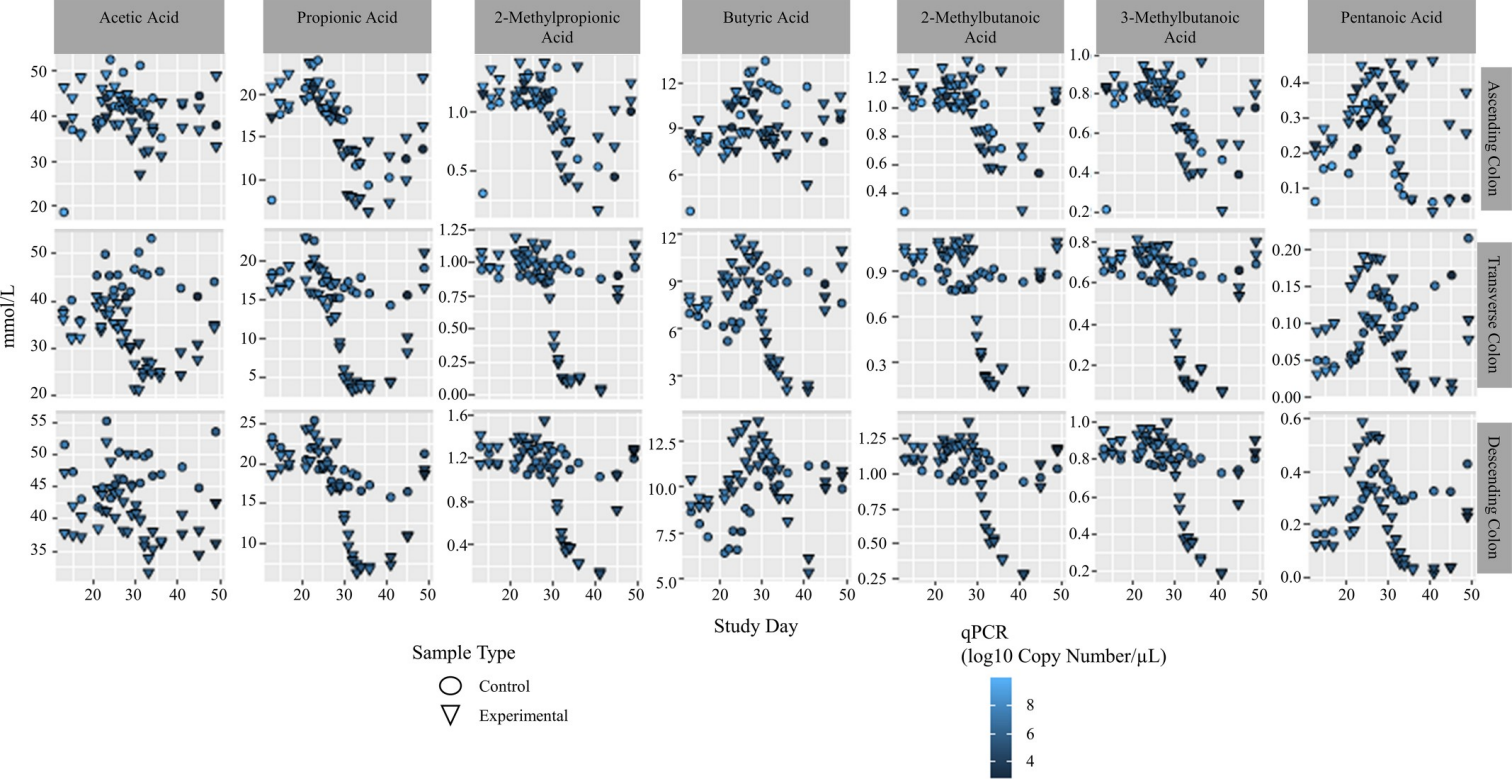
Short chain fatty acid data combined with copy number (qPCR). Top plots show the ascending colon, middle plots are the transverse colon, bottom plots are the descending colon. Copy number data is shown via a blue color gradient.

Short-chain fatty acids (SCFAs) are produced by the gut microbiota and absorbed by the colon cells, as well as used by other members of the bacterial community. Therefore, a change in bacterial composition may also change the production of SCFAs. Here, we looked at the concentrations of the most prominent 7 SCFAs in each of the 3 colon regions over the course of the experiment; acetic acid, propionic acid, 2-methylpropionic acid, butyric acid, 2-methylbutanoic acid, 3-methylbutanoic acid, and pentanoic acid. We combined this data with copy number data to show how the density of the bacterial community correlates with the SCFA concentration over the course of the experiment (Fig 6).

We found that overall, a large drop in SCFA concentration coincided with a drop in bacterial density due to exposure to a high dose of TCS. As seen in Fig 6, this is true for all analyzed SCFAs in both TC and DC. Where this appears to diverge is in the AC regions. In the AC regions, there is a similar decrease in SCFAs in the control samples along with the experimental samples during the High Dose treatment period with no corresponding decrease in bacterial density. The only SCFAs where this decrease does not occur are acetic acid and butyric acid.

**Fig 7:**
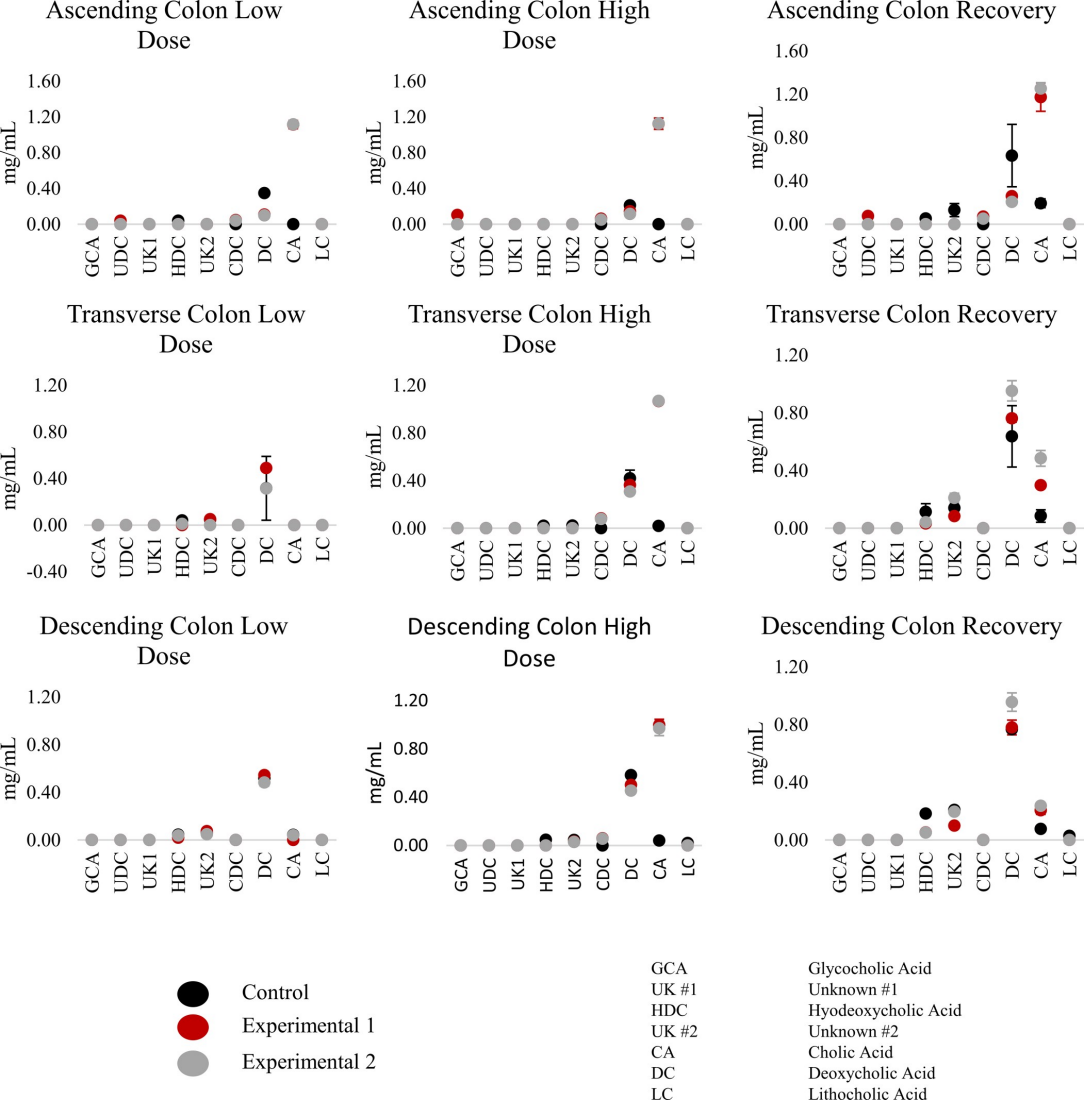
Bile acid concentration at the end of each experimental phase. Error bars are based on standard deviation.

Similarly, the conjugation and conversion of bile acids changed significantly in response to TCS, likely due to the smaller bacterial population (Fig 7). This is most clear in terms of the concentration of cholic acid (CA), where the exposure to the low dose of TCS results in a concentration similar to that of an untreated PJ/DM control (S7 Fig), especially in the AC. In the TC and DC regions, however, there is not a significant change in the concentration of CA and Deoxycholic acid (DCA) compared with control until the high dose experimental phase. Since intestinal microbes have previously been shown to perform a dihydroxylation reaction to form DCA, we infer that the lower bacterial population is the reason for this change in bile acid concentration [37, 38].

### Peak and trough analysis of selected bacterium

GRiD analysis of the samples sent for shotgun sequencing was performed to estimate the growth rates of 20 selected bacteria. The growth rates of the two experimental replicates were combined and compared to the growth rates of the control group (S6 Fig). For these plots, a high negative correlation suggests that treatment with TCS has an adversary effect on growth rate patterns, whereas a high positive correlation suggests that TCS treatment does not affect those patterns. Due to low sample numbers, none of the differences in growth rates shown here are statistically significant. For some of these bacterial species, however we do see some interesting patterns. For *Escherichia coli*, in all three colon regions, the growth rate of the experimental groups increased during the high dose period but decreased during recovery; however the high positive correlation on the AC suggests that the treatment does not alter its growth pattern. *Bacteroides thetaiotaomicron* has the opposite trend with a decreased growth rate during the high dose phase and an increase during the recovery time. In S6 Fig, *Escherichia coli*, *Citrobacter Koseri*, *Enterobacter cloacae*, *Citrobacter rodentium* and *Salmonella enterica* show an increase in growth rate in response to low and high treatment dosage. We observed a decrease in growth rate during treatment with *Bacteroides fragilis*, *Bacteroides thetaiotaomicron*, *Bacteroides vulgatus*, and *Parabacteroides distasonis*.

## Discussion

The widespread use of the antimicrobial TCS in products a consumer would use topically has already been banned [1]. So far, the use of TCS in toothpaste and other non-topical uses has not been banned in the U.S. However, there is strong evidence linking TCS to changes in gut microbial community and colonic disease in lesser animals [26–28, 39]. Two recent studies that addressed the impact of TCS from personal care products on the human gut microbial community *in vivo* found that use of TCS did not significantly impact the gut microbial diversity in adults or infants, though small changes were observed in specific members of the community [40, 41]. Here, we sought to answer whether the addition of TCS will impact the human gut microbial community *in vitro*. Using the SHIME® system allowed us to simulate the human gut microbial community *in vitro*, and to look at the community in the three different regions of the gut as well as in both the lumenal and mucosal phases.

In this study, we exposed a human gut microbial community to a low-dose and high-dose of TCS for a 1 week period. We used an artificially higher amount than what a normal exposure dose would be for the high dose for two reasons. First, our donor sample was obtained from a human who was very likely to have already been exposed to TCS over the normal course of their life, so we needed to raise the dose to ensure it would have an impact. Second, to ensure no important changes are missed at the low dose due to small, incremental change that may occur. Through this study we have shown that changes in the gut microbial community in response to TCS occur in a dose-dependent manner, and that these changes occur most strongly in the AC region. This difference in colon region response is likely because TCS was injected into the AC bioreactors. Looking at beta diversity, we do not show a complete recovery to the diversity present in the control in either the lumenal or mucosal phases, though in some cases (AC, lumenal phase) it does approach control. It is possible that we could see those conditions converge if given more than 2 weeks of recovery time. However, it is also possible that they would never reach the same levels of diversity as control again.

Those changes exist with respect to the community structure, changing from a *Bacteroidetes* and *Firmicutes* dominant community to one that is dominated by *Synergistetes*, *Fusobacteria*, and *Proteobacteria* (S2 Fig). Reduction of *Bacteroidetes* and *Firmicutes* abundance in the gut microbial community is associated with increased IBD in the westernized human population, and a difference in the ratio of *Bacteroidetes*:*Firmicutes* is associated with, but not prescriptive of, increased obesity [42]. Some types of *Fusobacteria*, the genus that increased in response to TCS, have been linked to colorectal cancer [43]. However, after the recovery period none of these genera were significantly different from the control group when using 16S sequencing data.

A determining factor regarding the type of bacteria impacted by TCS was those that decreased in proportional abundance are obligate anaerobes, and those that increase in the presence of TCS are facultative anaerobes. It has been speculated in soil microbial communities that TCS may act as an electron acceptor for particular bacterial species, which may explain this effect [44]. We also showed a decrease in diversity within samples over the course of the experiment, especially at the high dose treatment level. Perhaps most interestingly, we showed that the decrease in community diversity, structure, SCFA production, bile acid conversion and bacterial population following High Dose TCS treatment is close to recovery within the 2 week recovery period in the lumenal phase samples, however, it does reach recovery levels in the mucosal phase samples. The ability of the community diversity to climb back towards that of the stabilization period, but not quite reach it could be for a couple reasons: the first, that two weeks was not a long enough recovery time, and given enough time the community may reach pre-treatment levels of diversity again; the second is that the depletion of the bacterial community is not fully recoverable due to other strains taking precedence in the community after treatment with TCS. This second reason is particularly concerning because previous work has shown that a lack of diversity in the gut microbiota is not recoverable once it has been passed on mother to offspring [45]. If this diversity has been lost due to human exposure to TCS over the past several decades, this could have serious health effects.

There are some limitations to this experiment. The first major limitation is that any starting material for this type of experiment is someone who has been exposed to TCS in some form for most of their life. Previous research has shown that exposure to TCS over the course of the development of an anaerobic bacterial community has a profound impact of the structure of that community and the ability of it to develop antimicrobial resistance [46, 47]. TCS has also been shown to aid in formation of biofilms of *Staphylococcus aureus* in the nasal passages of a murine mouse model [48]. Along with the natural protective effects of biofilms, this may explain why our mucosal bacterial samples changed much less rapidly with the addition of TCS, and did not recover to the same extent in the two week recovery period (S3 Fig). Secondly, in a normal human body, the SCFAs would be absorbed and interact with the human cells that line the gut. A future experiment to explore how this change in metabolite availability would impact those human cells would be helpful. It would also be interesting to see how a recovery period would impact the inflammation typically seen in the mouse gut with *in vivo* TCS studies.

A decrease in gut microbial community diversity, also a hallmark of a westernized diet, is linked with increased incidence of IBD [49]. This study does reinforce the concern that the use of antimicrobials in many consumer products may be contributing to the population impacted by IBD by decreasing types of bacteria associated with a healthy gut (*Bacteroidetes*), changing the composition of the gut microbial community, and decreasing the gut microbial population as a whole. Since TCS has also been shown to decrease effectiveness of antibiotics in humans by increasing the frequency of persister cells, as well as its correlation with reproductive defects, its use in consumer products could be considered a danger to public health beyond its impact on the gut microbial community [10, 11]. Considering the persistent and high concentrations in residual biosolids from waste water treatment plants and the subsequent use of those biosolids as part of fertilizer and soil enhancement technologies on crops that are consumed by humans, it is becoming increasingly imperative that we understand the impact of TCS consumption on all aspects of human health. Here we demonstrated that its negative impact may be reversible, provided it can be removed from consumption entirely.

## Supporting information

S1 Fig Experiment diagram: Diagram of the Simulator of the Human Intestinal Microbial Ecosystem (SHIME®) in the Triple SHIME® configuration. (TIF)

S2 Fig Bacterial composition of lumenal samples based on proportional abundance. Taken from 16S rRNA data, shown at the family, genus level.
(TIF)

S3 Fig Bacterial composition of mucosal samples based on proportional abundance. Taken from 16S rRNA sequencing data, shown at the family, genus level.
(TIF)

S4 Fig Gene proportion heat map from shotgun sequencing data. (TIF)

S5 Fig Enoyl-acyl carrier protein gene analysis based on shotgun data. (TIF)

S6 Fig Growth rate obtained from GRiD analysis. (TIF)

S7 Fig Bile acid analysis of PJ/DM: Bile acid analysis of the pancreatic juice/ defined medium (PJ/DM) control. (TIF)

S1 Table 16S rRNA data: In this table, 16S rRNA sequencing data was used to determine the community members for each sample and grouped into Operation Taxonomic Units (OTUs). On the left, the phylogeny for each OTU is listed, and the relative abundance proportion for each OTU in the sample sequenced is given.
(XLSX)

S2 Table Shotgun sequencing data: In this table, shotgun sequencing data was used to determine the community members for each sample and grouped into OTUs. The phylogeny for each OTU is listed on the left, and the relative abundance proportion for each OUT in the sample sequenced is given.
(XLSX)

S3 Table qPCR data: In this table, the copy number per μL of DNA was determined in each sample. (XLSX)

S4 Table SCFA data: In the table provided, the short chain fatty acid (SCFA) measurements for each sample are given, as well as the averages for the samples, and ratios of Acetate:Proprionate:Butyrate for each sample. (XLSX)

S5 Table Bile acid analysis data: In the tables provided, the bile acid measurements for each sample are recorded and averaged. (XLSX)
