# Supplementary figures and images for: Triclosan has a robust, yet reversible impact on human gut microbial composition in vitro
Source: PLoS One. 2020 Jun 25;15(6):e0234046. doi: 10.1371/journal.pone.0234046 (PMC7316517; doi:10.1371/journal.pone.0234046)

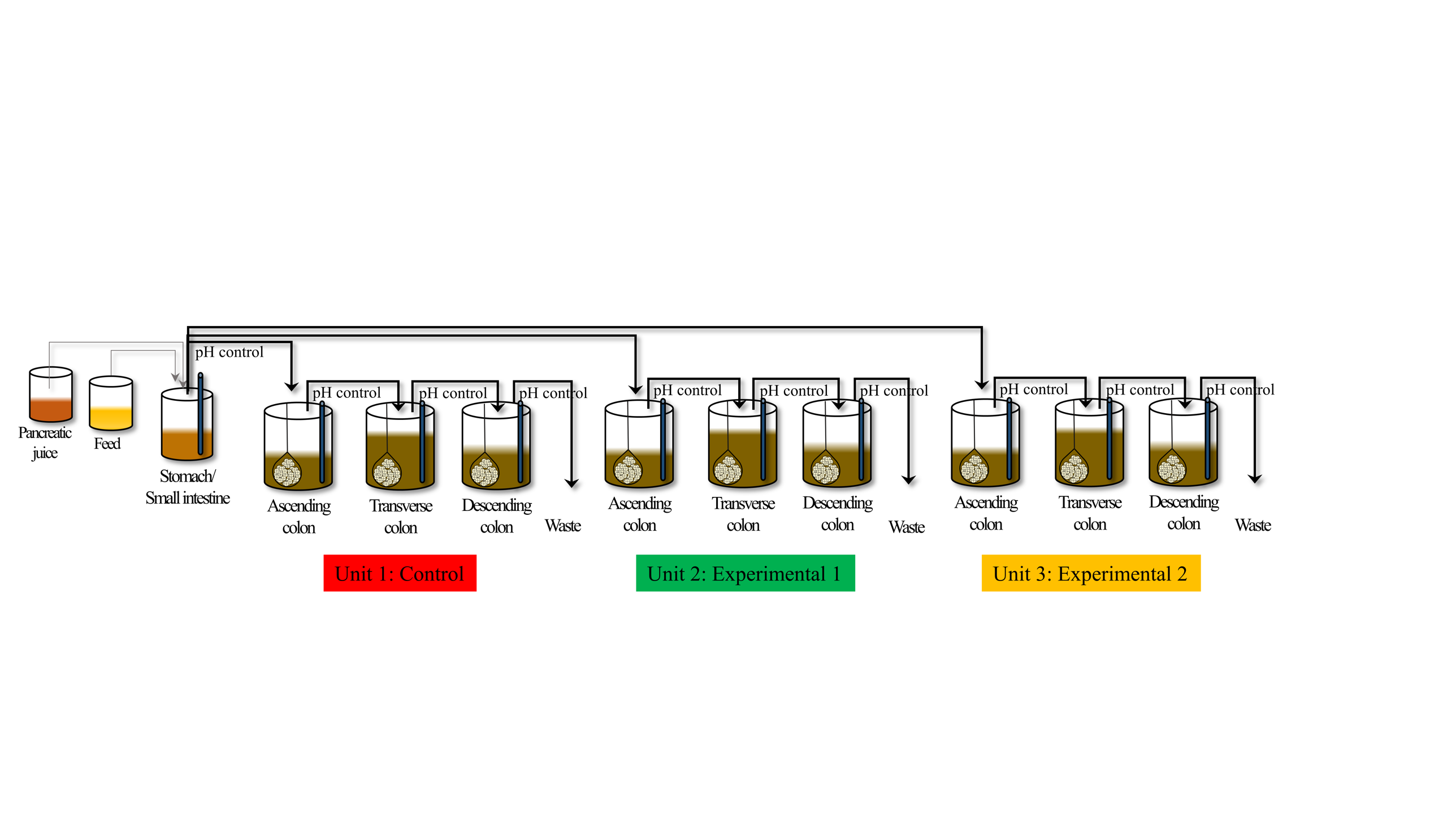

Supplement: S1 Fig — (TIF) [file pone.0234046.s001.tif]

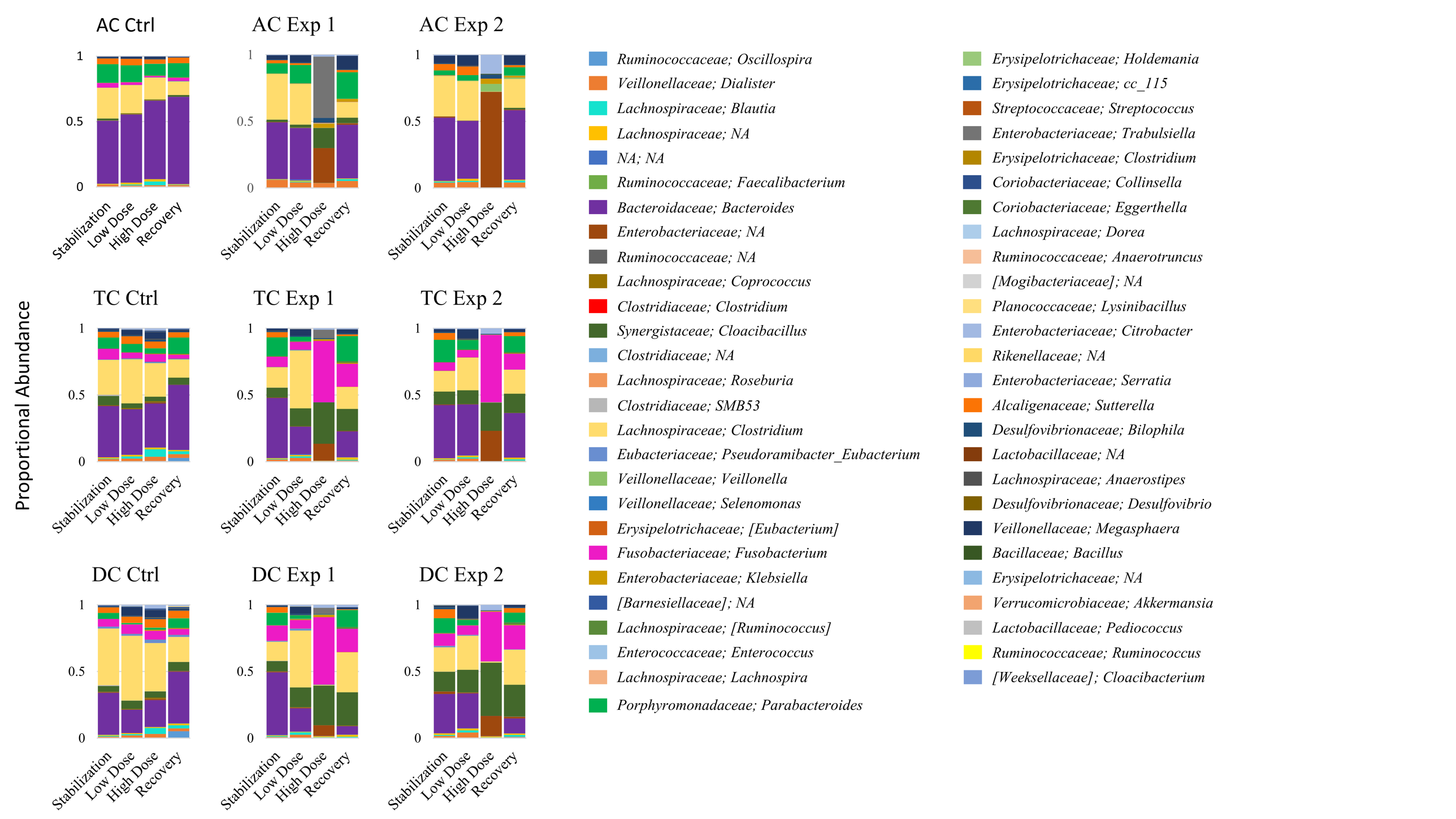

Supplement: S2 Fig — Taken from 16S rRNA data, shown at the family, genus level. (TIF) [file pone.0234046.s002.tif]

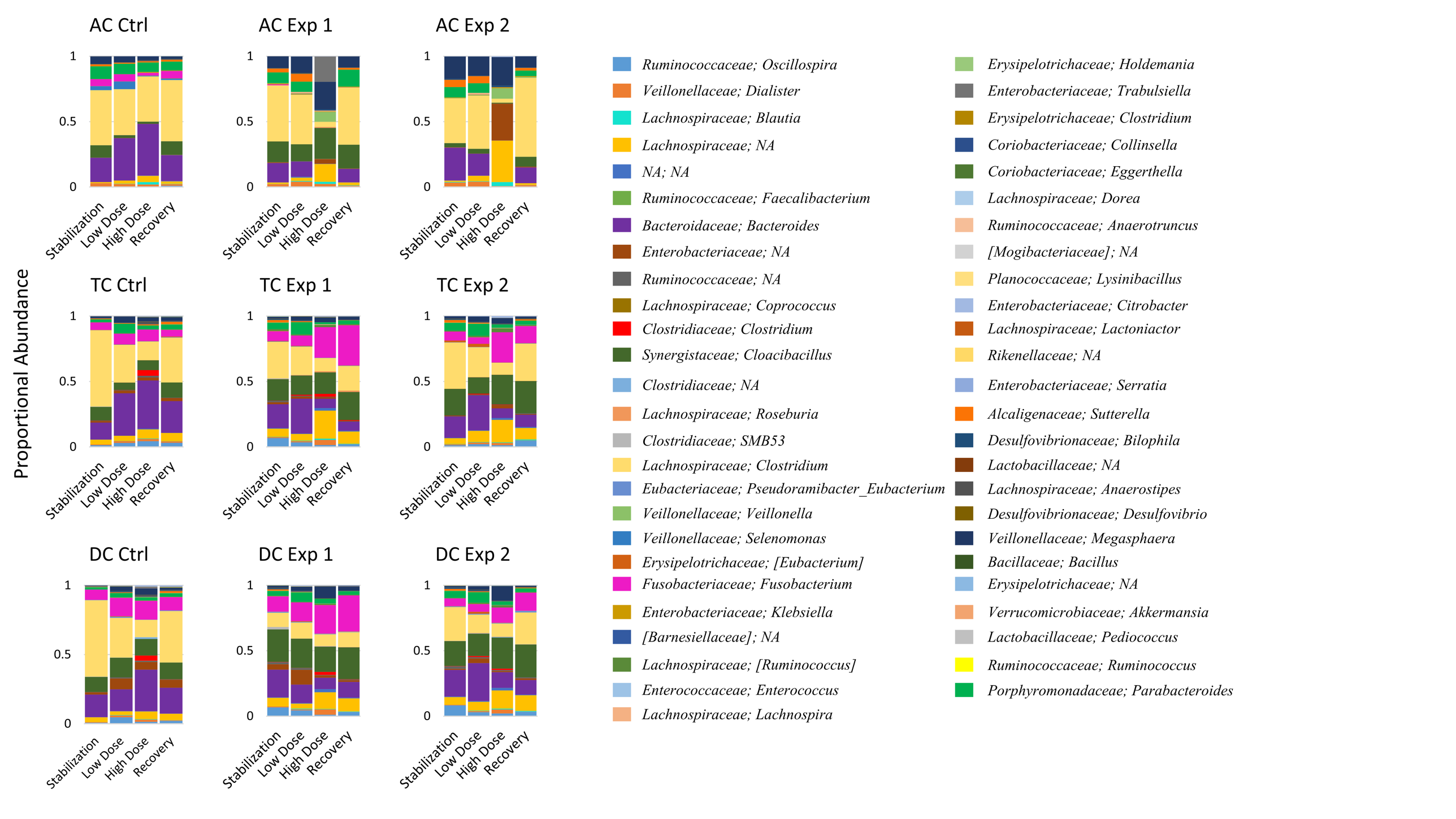

Supplement: S3 Fig — Taken from 16S rRNA sequencing data, shown at the family, genus level. (TIF) [file pone.0234046.s003.tif]

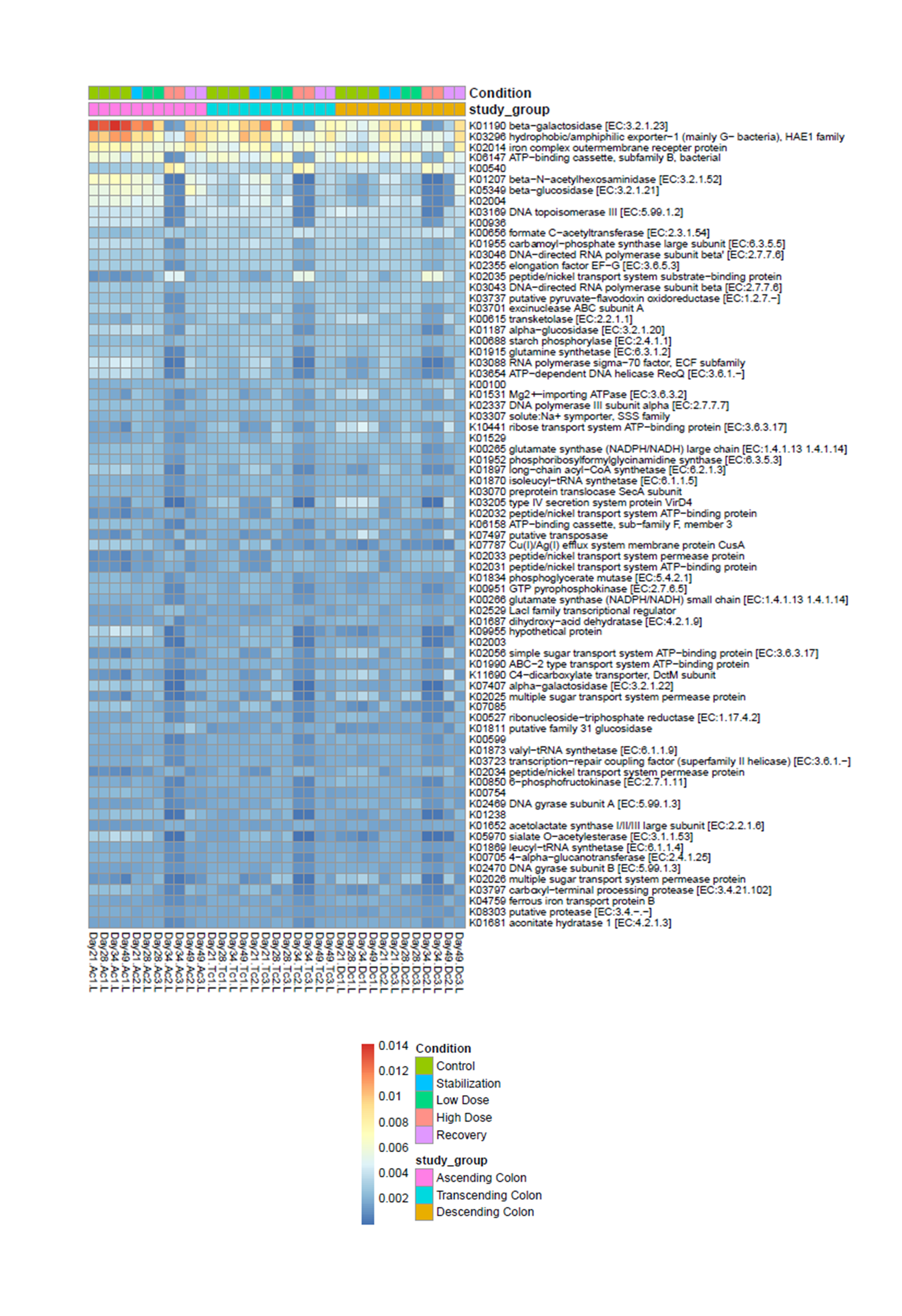

Supplement: S4 Fig — (TIF) [file pone.0234046.s004.tif]

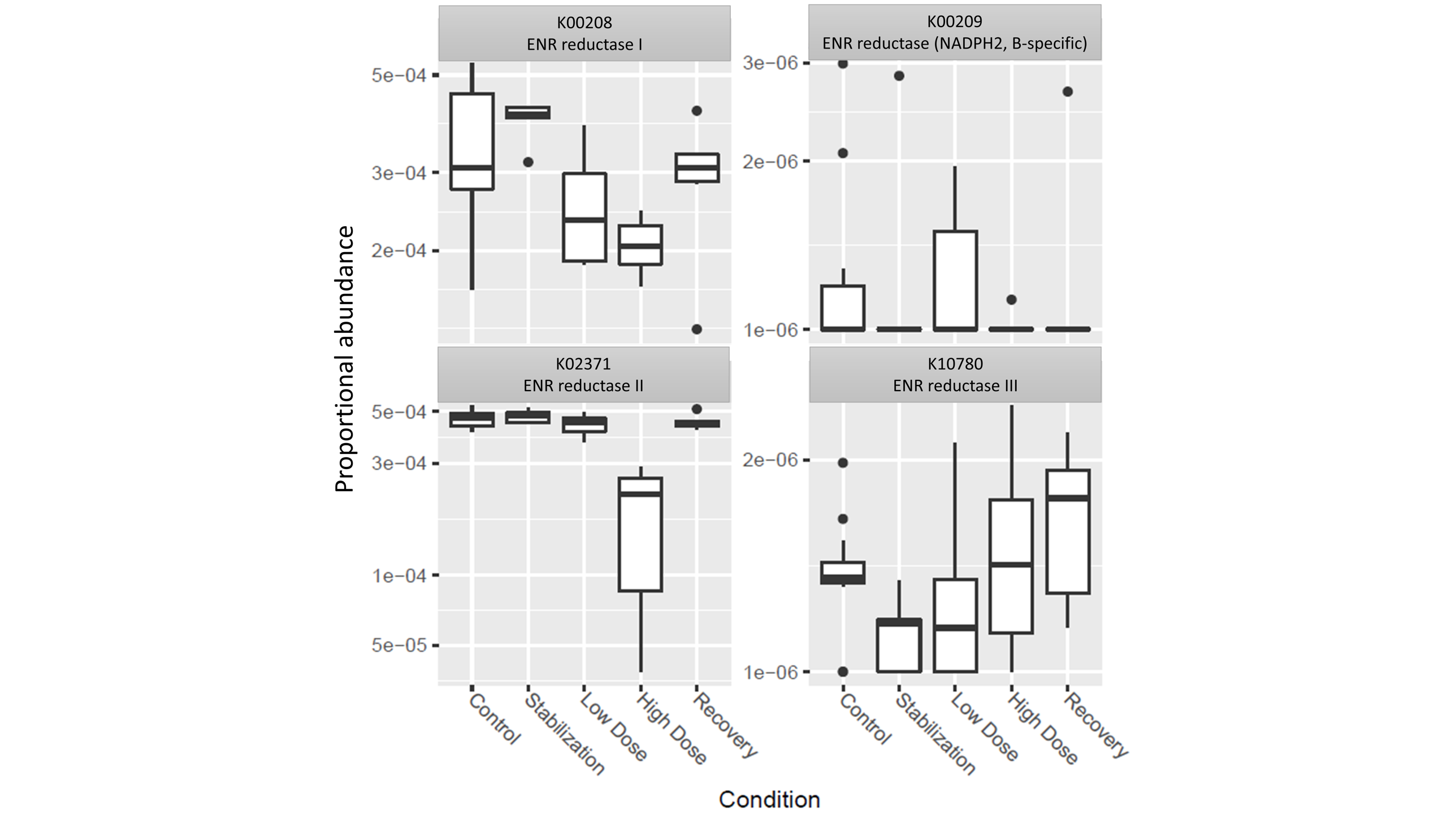

Supplement: S5 Fig — (TIF) [file pone.0234046.s005.tif]

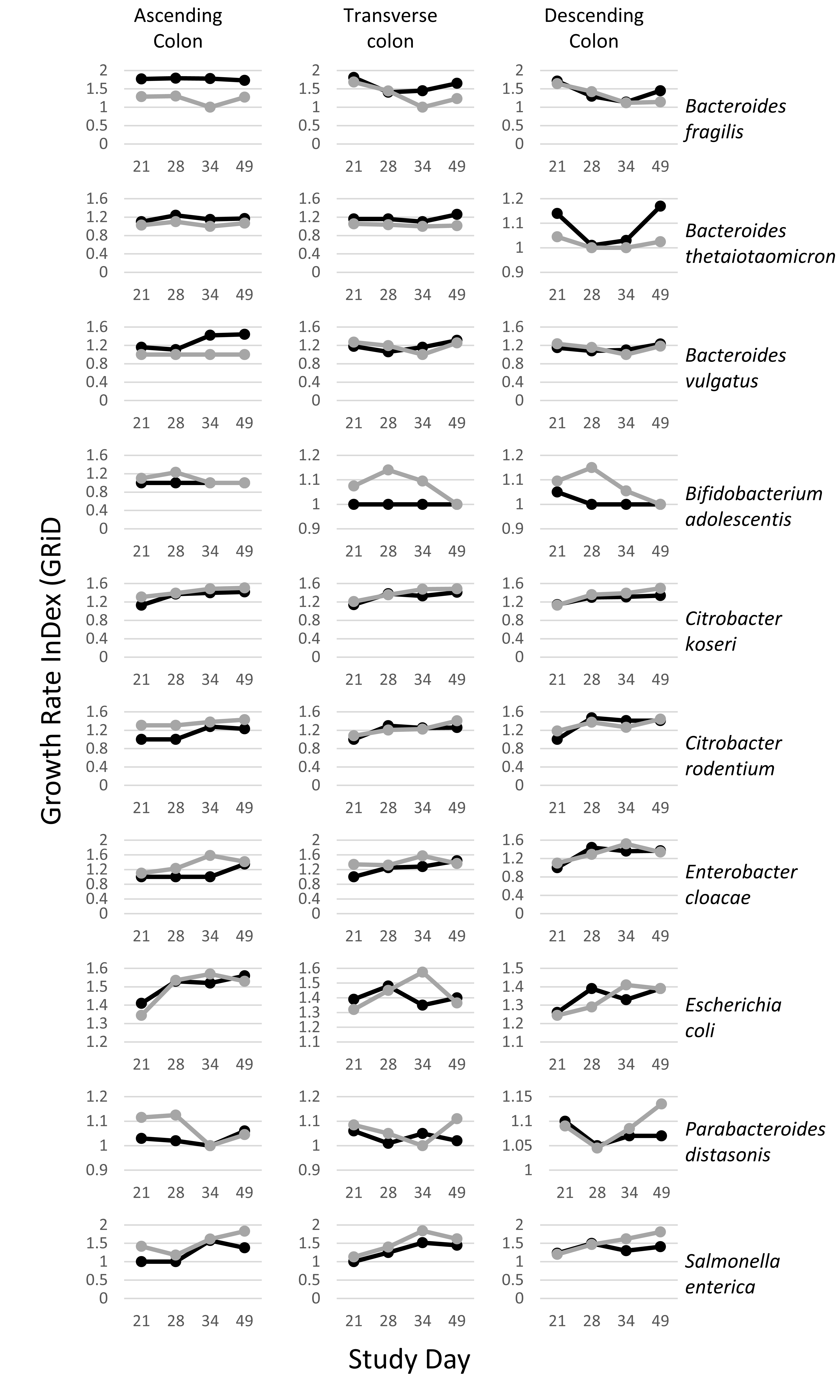

Supplement: S6 Fig — (TIF) [file pone.0234046.s006.tif]

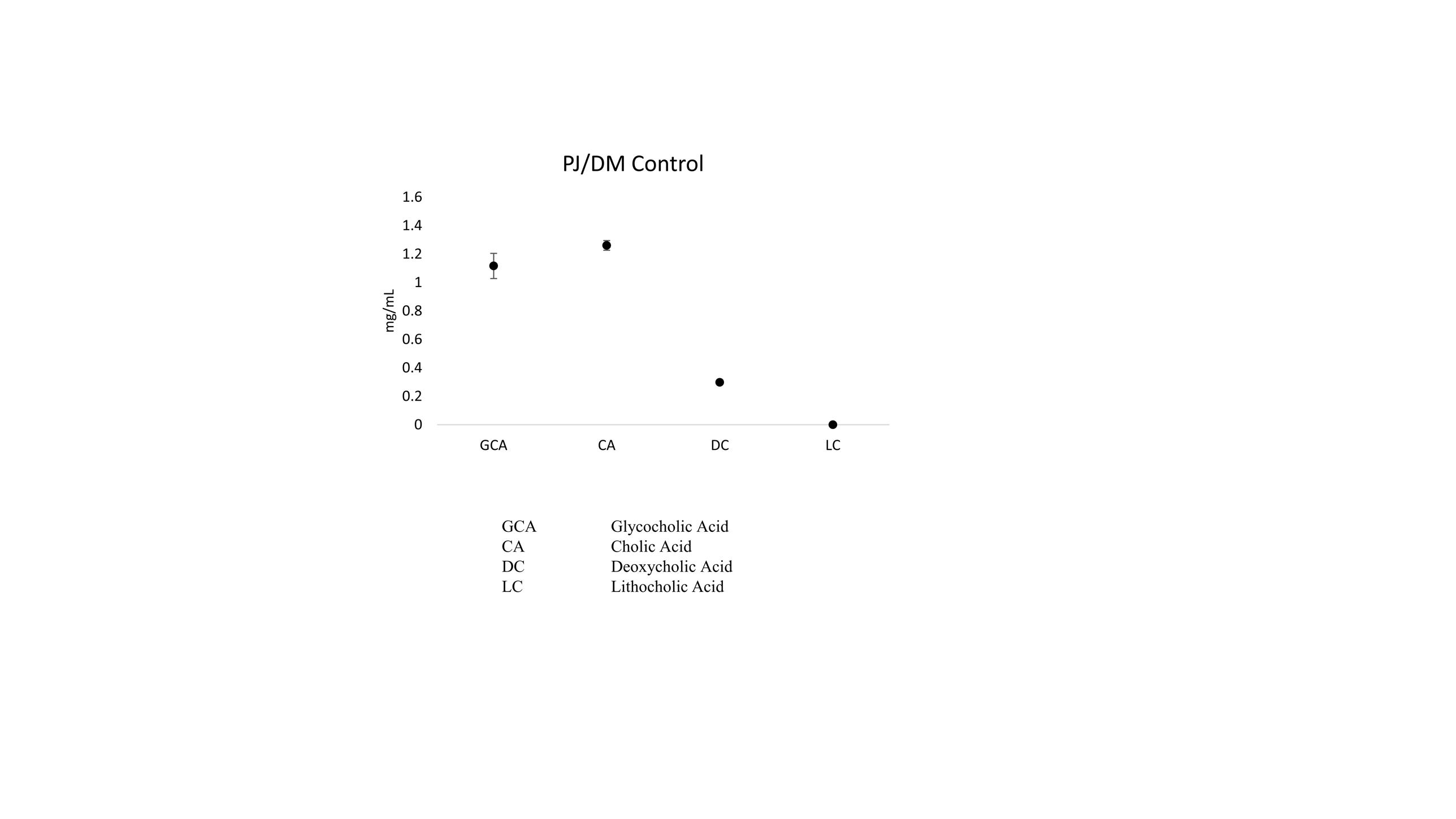

Supplement: S7 Fig — (TIF) [file pone.0234046.s007.tif]
